# Case Report: A rare case of primary hepatic Castleman’s disease mimicking a liver tumor

**DOI:** 10.3389/fonc.2022.974263

**Published:** 2022-08-30

**Authors:** Hong Chen, Xiaoxi Pang, Jie Li, Baixuan Xu, Yachao Liu

**Affiliations:** ^1^ Department of Nuclear Medicine, The Second Hospital of Anhui Medical University, Hefei, China; ^2^ Department of Pathology, Chinese PLA General Hospital, Beijing, China; ^3^ Department of Nuclear Medicine, Chinese PLA General Hospital, Beijing, China

**Keywords:** unicentric castleman’s disease, mri, pet/ct, liver, pathology

## Abstract

Castleman’s disease (CD) is a primary lymphoproliferative disorder of the lymph nodes with rare extra-nodal primary affection. Solid organ involvement is rare, and isolated liver involvement is extremely rare. Here we presented a case of a 59-year-old woman with a hepatic lesion accidentally found by ultrasound. The MRI result indicated primary liver malignancy or liver metastases. ^18^F-FDG PET/CT could not exclude hepatic malignant tumor due to its high metabolism. Finally, the hepatic CD was confirmed by postoperative pathology.

## Background

Castleman’s disease (CD) is a lymphoproliferative abnormality, also referred to as giant lymph node hyperplasia or follicular lymphoid hyperplasia. CD can occur wherever lymphoid tissue is found. Clinically, it occurs as a localized unicentric CD (UCD) or as a systemic multicentric CD (MCD) (1). Most cases of UCD are asymptomatic and diagnosed accidentally by imaging. A small number of patients may have symptoms of mass effect (2). Radiological diagnosis of hepatic CD by computed tomography (CT) or magnetic resonance imaging (MRI) remains difficult. The lack of characteristic clinical and radiological features makes pathological examination the gold standard for the diagnosis of hepatic CD. Surgical resection has proven to be curative for UCD.

## Case presentation

**Figure 1:**
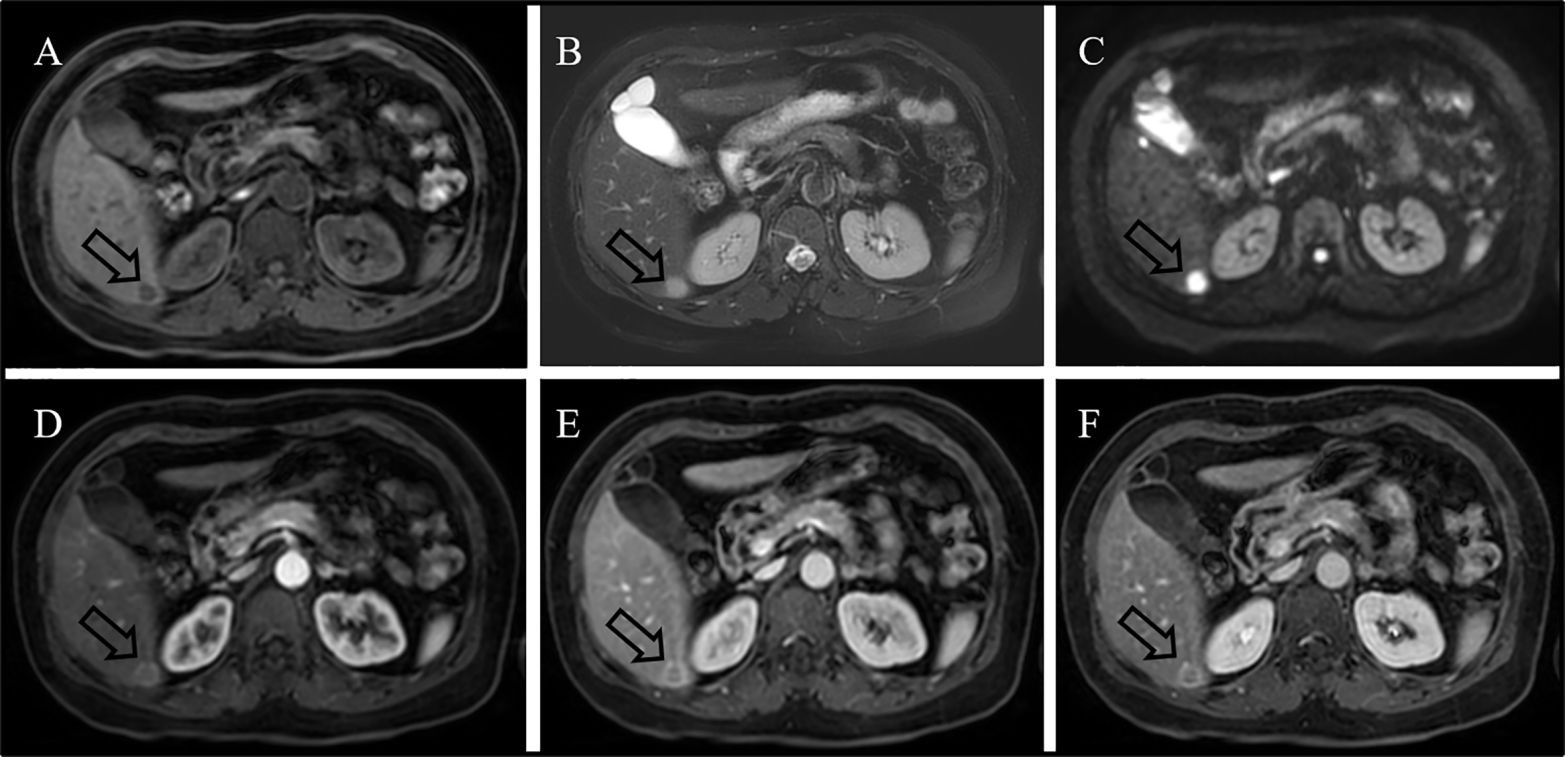
MRI result showing long T1 [**(A)**, arrow] and slightly longer T2 [**(B)**, arrow] signals with a diameter of 1.5 cm in the S6 segment of the liver. Diffusion-weighted imaging result showing a high signal [**(C)**, arrow]. Enhanced scanning result showing slight circular enhancement in the arterial phase [**(D)**, arrow] and continuous circular enhancement in the portal venous phase [**(E)**, arrow] and delayed phase [**(F)**, arrow].

**Figure 2:**
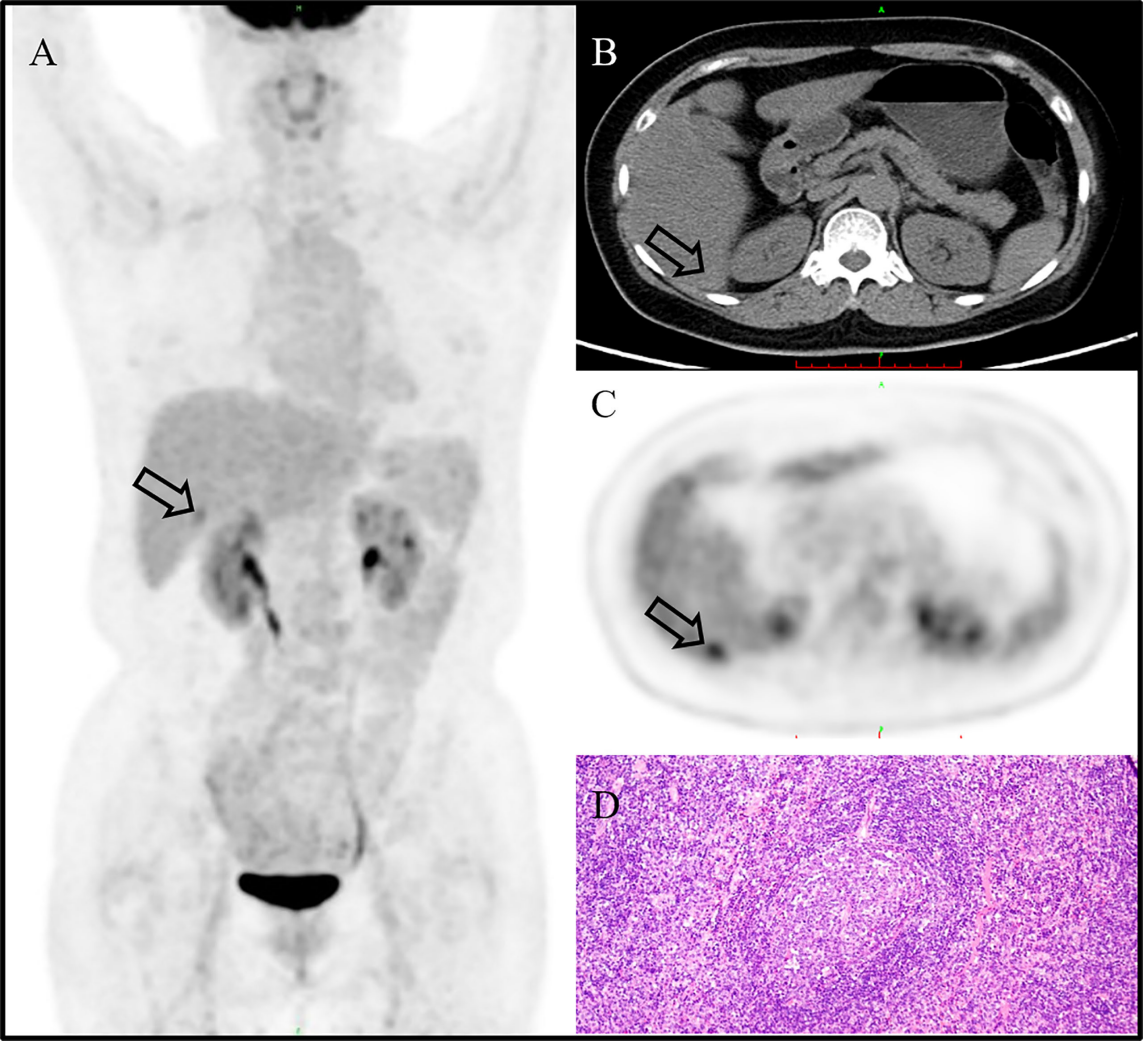
^18^F-FDG PET/CT image showing no other abnormal hypermetabolic lesion except in the liver [**(A)**, MIP, arrow]. The liver lesion could not be clearly displayed in the background of fatty liver on CT [**(B)**, CT image, arrow]. The CT mean value of the liver was about 37 HU. The lesion showed a high uptake of ^18^F-FDG [**(C)**, PET image; arrow], and the SUVmax is 4.0. It was finally confirmed as Castleman’s disease by postoperative pathology [**(D)**; H&E, ×200].

A 59-year-old woman without any symptom was accidentally presented with a space-occupying lesion in the liver by abdominal ultrasound. The patient had no history of hepatitis B, and the routine blood examination showed that the white blood cells were 5.46 × 10^9^/L, the red blood cells were 3.89 × 10^12^/L, hemoglobin was 120 g/L, and the platelets were 274 × 10^9^/L. The tumor markers including carcinoembryonic antigen, alpha-fetoprotein, and CA19-9 were also within the reference range. Ultrasonography showed a very hypoechoic nodule with a clear boundary of about 1.3 cm × 1.6 cm in the S6 segment of the liver. The MRI (**Figure 1**) result showed that the liver was normal in size and shape, with a diameter of 1.5-cm-long T1 and a slightly longer T2 signal nodule in the S6 segment of the liver. The diffusion-weighted imaging result showed a slightly higher signal. Enhanced scanning showed slight circular enhancement in the arterial phase and continuous circular enhancement in the portal venous phase and delayed phase. The MRI result suggested the possibility of primary liver malignant tumor or liver metastasis.^18^F-FDG PET/CT (**Figures 2A–C**) showed no other abnormal hypermetabolic lesion except the liver. According to the above-mentioned examinations, the surgeon could not rule out the possibility of liver malignant tumor. Twenty days later, the patient underwent laparoscopic resection of the tumor. The intraoperative bleeding was about 20 ml. There were no postoperative complications, and the patient was discharged 3 days later. The postoperative pathology showed that the size of the liver lesion was about 1.7 cm × 1.5 cm × 1.5 cm, and HE staining (**Figure 2D**) showed lymphoproliferative lesions, including multiple follicular structures, some follicular germinal centers, marginal zone cell proliferation, mutual encirclement and fusion, and proliferation of small vessels with hyaline degeneration of the walls in the follicles. The liver lesion was finally confirmed as Castleman’s disease by postoperative pathology.

## Discussion and conclusion

CD is a nonmalignant lymphoproliferative disorder that, although uncommon, is a relatively complex disease to diagnose and manage (3). UCD occurs most commonly in the mediastinum, cervical regions, and abdominal/pelvic cavity but can be found in any lymph node station (1). Rarely, UCD occurs in the lung (4), kidney (5), orbit (6), parotid gland (7), spleen (8), accessory spleen (9), small intestine (10), pancreas (11), porta hepatis (12), and adrenal gland (13). However, a focal Castleman’s disease located in the liver is extremely rare.

UCD occurs most frequently as a focal lesion that is not associated with obvious clinical symptoms and is often incidentally found during routine physical examination. In our case, the patient was accidentally found to have a space-occupying lesion in the liver by abdominal ultrasound without any symptom, and no obvious positive findings were found in the relevant laboratory tests.

CD often shows well-defined and mildly hypodense or isodense homogeneous masses on nonenhanced CT/MRI, with intermediate and marked enhancement on contrast-enhanced CT/MRI (14). In our case, the hepatic CD showed slight circular enhancement in the arterial phase and continuous circular enhancement in the portal venous phase and delayed phase. Whole-body PET/CT has a role to assess the metabolic status of lymph nodes and extra-nodal lesions. On ^18^F-FDG PET/CT, CD showed an increased uptake of varying degrees, with SUVmax that ranged from 2 to 19 (4, 15). In our case, primary hepatic CD also presents an increased uptake of ^18^F-FDG, and the SUVmax is 4.0. In view of its high metabolism, it is easy to be misdiagnosed as malignant tumor.

A definitive diagnosis of CD is usually based on histopathological examination. The main pathological features of CD are lymphoid follicular hyperplasia and vascular vitreous degeneration. Histopathologically, it can be divided into hyaline vascular, plasma cell, and mixed variants (16). Most cases of UCD have been categorized pathologically as being of the hyaline vascular type (2), as is our case. The treatment of choice for UCD is surgical resection, which is associated with 90% relapse-free survival (3).

In conclusion, UCD originating in the liver is extremely rare and should be considered in the differential diagnosis of a primary or secondary malignancy of the liver.

## Data availability statement

The raw data supporting the conclusions of this article will be made available by the authors, without undue reservation.

## Author contributions

All authors listed have made a substantial, direct, and intellectual contribution to the work, and approved it for publication.

## Funding

This work was supported by Anhui Provincial Natural Science Foundation (2008085QH406) and Clinical Research Cultivation Program of The Second Affiliated Hospital of Anhui Medical University (2021LCYB08).

## Conflict of interest

The authors declare that the research was conducted in the absence of any commercial or financial relationships that could be construed as a potential conflict of interest.

## Publisher’s note

All claims expressed in this article are solely those of the authors and do not necessarily represent those of their affiliated organizations, or those of the publisher, the editors and the reviewers. Any product that may be evaluated in this article, or claim that may be made by its manufacturer, is not guaranteed or endorsed by the publisher.
